# A Compend on the Diseases of the Eye and Refraction, Including Treatment and Surgery

**Published:** 1899-10

**Authors:** 


A COMPEND ON THE DISEASES OF THE EYE AND REFRACTION, INCLUDING TREATMENT AND SURGERY. By Geo. M. Gould, A.M., M.D., and Walter L. Pyle, A.M, M.D. Published by P. Blakiston's Son & Co., Philadelphia. Price 80 cents.

This is one of the Quiz-Compend Series, gotten out by the well-known publishing house. These Compends have long been known to medical students, and have probably been a great aid to them in preparing for examinations. This little work is quite complete and contains throughout its pages well-recognized illustrations. Dr. Gould, as is to be expected, goes much into the refractive work, giving of course prominence to his special views on muscular anomalies. The book, however, is a safe guide to students. R.

